# Single-sex schistosome infections of definitive hosts: Implications for epidemiology and disease control in a changing world

**DOI:** 10.1371/journal.ppat.1006817

**Published:** 2018-03-01

**Authors:** Da-Bing Lu, Yao Deng, Huan Ding, You-Sheng Liang, Joanne P. Webster

**Affiliations:** 1 Department of Epidemiology and Statistics, School of Public Health, Soochow University, Suzhou, China; 2 Jiangsu Key Laboratory of Preventive and Translational Medicine for Geriatric Diseases, School of Public Health, Soochow University, Suzhou, China; 3 Key Laboratory of National Health and Family Planning Commission on Parasitic Disease Control and Prevention, Wuxi, China; 4 Jiangsu Provincial Key Laboratory on Parasites and Vector Control Technology, Wuxi, China; 5 Jiangsu Institute of Parasitic Diseases, Wuxi, China; 6 Centre for Emerging, Endemic and Exotic Diseases (CEEED), Department of Pathology and Population Sciences, Royal Veterinary College, University of London, London, United Kingdom; University of Pittsburgh, UNITED STATES

## Introduction

A substantial proportion of the world’s disease burden is caused by infectious agents that lead to high mortality, morbidity, and reduced productivity among many millions of people and their animals. Guided by the Millennium Development Goals (MDGs) and the subsequent Sustainable Development Goals (SDGs), much progress has been made in reducing the burden of human infectious diseases since 2000 [1], although challenges remain and new questions emerge.

Human schistosomiasis is a neglected tropical disease (NTD) caused by blood flukes of the genus *Schistosoma*. The parasitic disease remains a public health problem in the majority of 78 tropical and subtropical countries, with approximately 261 million people in need of treatment [2]. After nearly 70 years of major multidisciplinary control efforts, great success has been achieved against *Schistosoma japonicum* within China. Across sub-Saharan Africa, efforts to control *S*. *mansoni* and *S*. *haematobium* through large-scale preventive chemotherapy (PC) with praziquantel (PZQ) have also had a substantial impact on preventing or relieving morbidity and improving global health, particularly amongst the poorest [3]. The success of such activities has in part led to a revision of WHO’s strategic plan and a vision for ‘a world free of schistosomiasis’, with elimination as a public health problem and complete interruption of transmission in selected regions by 2025 [4].

However, recent reports of prevalence levels greater than previously thought [5], re-emergence of schistosomiasis in previously controlled regions [6], and emergence of zoonotic hybrid *Schistosoma* within previously uninfected regions [7], as well as indications of reduced drug efficacy amongst populations under high PC pressure [8], all serve to highlight that schistosomes are highly complex multi-host parasitic organisms. Many essential characteristics of their biology and epidemiology remain unknown.

## Single-sex schistosome exposure and infection of humans: An overlooked phenomenon

Schistosomes are unique amongst the trematode flukes in that they are dioecious, with male and female worms pairing in either the hepatic or urogenital system, depending on the species. Their spined eggs becoming trapped within host tissues (with subsequent granuloma development) causes the primary pathogenicity. The phenomena of single-sex schistosome infection within definitive hosts is generally dismissed as relevant only in terms of its possibility for ‘egg-negative/worm-positive schistosomiasis’ in areas of otherwise low prevalence [5]. In such situations, people appear to harbor adult worms based on detection of worm antigens (e.g., through Point-of-care Circulating Cathodic Antigen [POC-CCA] or Point-of-care Circulating Anodic Antigen [POC-CAA] tests) but are not excreting eggs (as determined by Kato–Katz or urine filtration) (Box 1). There are, however, a number of reasons to predict both a high incidence of single-sex cercarial exposures and subsequent adult worm infections, with unforeseen implications for the epidemiology, evolution, and control of disease.

**Box 1. Why single-sex *Schistosoma* spp. infections have been overlooked**

1. No symptoms occur, because no schistosome eggs are produced within a final host.
2. They cannot be identified with traditional egg-based parasitological tests.
3. There is logistical difficulty in the confirmation of single-sex schistosome infection of definitive hosts. This is only possible in nonhuman animals through dissection or perfusion.
4. Even during dissection or perfusion, female worms are more easily overlooked than their male counterparts due to their differential morphology (the relative width of *S*. *haematobium* males and females is 0.8–1 mm versus around 0.25 mm; in *S*. *mansoni*, the same; and in *S*. *japonicum*, 0.5–0.55 mm versus around 0.3 mm).
5. Likewise, single females, whether never paired or following the loss of their male partner, are significantly shorter relative to paired females (e.g., single *S*. *japonicum* females measure ≤5.35 mm compared to ≥13mm for paired females [21]), making detection of single females even less likely.
6. There is a lack of awareness of the possibility of single-sex infections. With antibody detection in humans, the serological prevalence in endemic areas is often higher than the parasitological prevalence. The gap has been usually attributed to past exposure, cross-reactions or false positive reactions [33], rather than a possible single-sex infection.
7. Likewise, with antigen and/or nucleic acid detection, e.g., following PZQ, humans have been demonstrated to be egg negative but remain POC-CCA positive [5, 34, 35] and/or nucleic acid positive [36], which has been assumed to be evidence of *Schistosoma* breakdown products rather than a remaining single-sex infection.
8. Experimental studies of antigen production during single-sex infections have been conflicting. In vivo single-sex *S*. *mansoni* infections in mice have reported differences in the quantity of antigens produced between the sexes, with females lower than males [37], although in vitro, more antigens were detected from females than males [38]. Meanwhile, some in vivo single-sex *S*. *japonicum* studies in rabbits have not detected any circulating membrane antigen post-exposure [22].
9. There is a lack of recognition of the potential implications of current single-sex infections on subsequent transmission of schistosomiasis.

## Empirical evidence from the field and laboratory

Paired male and female schistosomes have a reported mean survival of 3–5 years [9], although there are documented cases (in humans living in nonendemic areas) of viable (i.e. egg-laying) schistosome pairs surviving several decades [10]. Experimental studies in laboratory hosts have reported similar infectivity profiles of both male and female cercariae following exposure [11]. However, it is the subsequent development and survival of single schistosomes that is generally considered to differ significantly between the sexes. It is well documented that male worms intimately control and regulate the expression of a number of female genes [12–14]. Conventional wisdom proposes that within a definitive host an unpaired male schistosome is able to mature, whereas an unpaired female cannot and remains immature [15, 16]. Furthermore, any unpaired female schistosomes (as a consequence of single-sex exposure and/or subsequent loss of a male partner) have been explicitly proposed to starve, with an inevitable short survival time [15, 17]. In accordance with this, some experimental studies have demonstrated a difference in male and female survival rates of 8 weeks post-single-sex exposure [11]. This, combined with the inherent logistical difficulties in identifying single-sex infections in the field (Box 1), has meant that single-sex infections are often dismissed as a dead end in terms of subsequent transmission and disease, particularly in the case of single females.

However, studies from both the field and the laboratory have reported multiple instances of single males and single females within infected hosts. An eight-year ecological survey on *S*. *mansoni* in wild rat (*Rattus rattus*) reservoir hosts in Guadeloupe revealed that amongst 207 infected rats of 503 captured, 164 harbored dual-sex adult schistosome pairs, whereas 43 rats harbored single-sex schistosome infections (38 of which were single adult males and five of which were single adult female worms) [18]. In 2010, along the Yangtze River within the Hubei province of China, among 400 sentinel mice used for detection of *S*. *japonicum* transmission sites, 22 mice were identified with schistosome infection, of which 14 were colonized with adult male schistosomes only and two with adult females only [19]. In experimental studies, when pigs were exposed to dual-sex combinations of *S*. *japonicum* cercariae, unpaired females were observed to survive up to 24 weeks [20]. Likewise, in single-sex schistosome infection experiments, female *S*. *japonicum* have been observed to survive 31 weeks in mice [21] and at least one year in rabbits [22], albeit on average at a smaller individual size and length than their paired female counterparts [21]. Such studies thereby provide convincing evidence that female schistosomes can exist unpaired in nature.

## Reproduction following single-sex infections

Experimental evidence has also demonstrated that single-sex schistosomes retain the ablity to successfully mate and reproduce if subsequent opportunities arise. In an experiment of pigs exposed to *S*. *japonicum* cercarial isolates at four-week intervals, male schistosomes of the first isolate were observed to have coupled with females of the second, suggesting that individuals unable to find a partner during the primary infection had successfully survived as single males and then paired when female partners became subsequently available [23]. Similarly, when 70-day-old female *S*. *mansoni*, derived from single-sex infections, were transferred to Nile rats together with mature male *S*. *mansoni*, all females were observed to have their vitelline glands developed up to the final stage at 11 days post-transference [24]. In our own laboratory, mice were initially exposed to only female *S*. *japonicum* cercariae, then five weeks later to only male *S*. *japonicum* cercariae. At six weeks post-second exposure, we recovered paired adult worms from the venous systems and eggs from liver tissues, further lending support to the evidence that single female schistosomes can survive over an extended period and subsequently viably reproduce when males become available.

## Impact of control and differential drug sensitivity

Under certain scenarios and with certain species, a majority of infected snails have been reported to harbor clonal single-sex infections (e.g., for natural infections of *S*. *japonicum* [25]). The natural low prevalence of infected snails in the field may enhance the likelihood of single-sex exposures to definitive hosts. Following PC, one may predict that the chances of single-sex infections may further increase, both at the individual and population level. Furthermore, in addition to the reduction in the circulating levels of cercariae in the environment from intermediate hosts due to increasing control measures, single-sex schistosomes have been demonstrated to be less sensitive to PZQ relative to sensitivity observed amongst dual-sex infections. In vivo laboratory mice with single-sex *S*. *mansoni* male infections at 7 weeks old were observed to have an ED_50_ (i.e., effective dose in reducing 50% worm burden) of PZQ 198 mg/kg, a significantly lower sensitivity to that of their dual-sex infection counterparts (PZQ 80.9 mg/kg). Moreover, single-sex females of the same age had an in vivo ED_50_ of 1,107 mg kg^-1^, requiring approximately 14 times the dose relative to that from dual-sex infections [26]. Furthermore, within the definitive host, since paired adult females reside within the groove of the males, male schistosomes may be predicted to be differentially exposed and succumb to the PZQ, leaving the surviving females intact [27]. It is likely, therefore, that single-sex schistosome infections and/or unbalanced dual-sex infections would differentially survive PC and subsequently pair and reproduce when the opportunity arises, thereby providing an additional explanation for the apparent lower PZQ cure/egg reduction rates observed under certain conditions [8, 28].

## Implications and applications

These studies raise a number of implications of potentially profound importance for elucidating schistosome transmission dynamics and our opportunities for control. The predicted common incidence of single-sex schistosome infections, their survival for longer than originally assumed, and an apparently lower drug sensitivity amongst single-sex infections could have a great influence on the epidemiology and evolution of the parasite. The definitive host may act as a ‘refugium’ for single-sex schistosomes, which might also possess genes related to infectiousness, virulence, or drug resistance. As *Schistosoma* spp. exposures often recur repeatedly over a host’s lifetime, it seems probable that initially single schistosomes may be able to pair and viably reproduce with subsequent arrivals of the opposite gender, recombining the genes. These consequences may be enhanced when the incomer is from a different generation, geographical location, or genotype. The unprecedentedly large scale of human migration to and from previously endemic areas would facilitate the above process. Furthermore, viable hybridization between different species of schistosome (particularly those between human and livestock *Schistosoma* species) is a major emerging public health concern at the intersection of infectious disease biology and evolution [29]. The ability of single-sex schistosome infections to be viably maintained within a human host (with pairing and reproduction with the opposite sex whenever available—even from a different species) may be another plausible explanation for the reported high levels of bidirectional viable pairings from Africa to Europe between *S*. *haematobium*, *S*. *bovis*, *S*. *currassoni*, and *S*. *intercalatum* [7, 30–32].

## Conclusions

Schistosomes are dioecious. Single-sex schistosome exposures and subsequent infections are predicted to become more common when the prevalence of the parasite in the environment decreases, as in response to recent increases in successful PC programmes. The likelihood of single-sex infections may be further enhanced by differential survival between sexes when a dual-infected host receives drug treatment. The existence of established and stable single-sex schistosome infections within definitive hosts and their potential for subsequent reproduction could be an emerging and profound threat to realization of the target for global schistosomiasis elimination [4]. Current recognition, combined with additional research into the frequency of this situation, the long-term viability of unpaired schistosomes of either gender, the potential for differential species-specific competitive success, and the implications of their existence for recent antigen (POC-CCA and POC-CAA) or nucleic acid detection diagnostics, is imperative if we are ever to fully understand the biology of this complex parasite and ultimately reach the proposed targets for schistosomiasis elimination.
